# High-Throughput Sequencing is a Crucial Tool to Investigate the Contribution of Human Endogenous Retroviruses (HERVs) to Human Biology and Development

**DOI:** 10.3390/v12060633

**Published:** 2020-06-11

**Authors:** Maria Paola Pisano, Nicole Grandi, Enzo Tramontano

**Affiliations:** 1 Laboratory of Molecular Virology, Department of Life and Environmental Sciences, University of Cagliari, 09042 Cagliari, Italy; mp.pisano@unica.it (M.P.P.); nicole.grandi@unica.it (N.G.); 2 Istituto di Ricerca Genetica e Biomedica, Consiglio Nazionale delle Ricerche, 09042 Cagliari, Italy

**Keywords:** HERVs, human genome, high-throughput sequencing, expression

## Abstract

Human Endogenous retroviruses (HERVs) are remnants of ancient retroviral infections that represent a large fraction of our genome. Their transcriptional activity is finely regulated in early developmental stages and their expression is modulated in different cell types and tissues. Such activity has an impact on human physiology and pathology that is only partially understood up to date. Novel high-throughput sequencing tools have recently allowed for a great advancement in elucidating the various HERV expression patterns in different tissues as well as the mechanisms controlling their transcription, and overall, have helped in gaining better insights in an all-inclusive understanding of the impact of HERVs in biology of the host.

## 1. Introduction

Human Endogenous Retroviruses (HERVs) are repetitive elements derived from infection by ancient retroviruses, now extinct, that integrated within the genome of primate germ line cells and have been transmitted as proviruses through the offspring [1]. These sequences currently constitute a significant portion (about 8%) of human DNA [2,3]. The majority of HERVs have lost their replication and expression activities, mostly due to deletions and recombination events, or for the accumulation of several mutations that inactivated them, while some HERVs have maintained the classical proviral structure—including the main genes flanked by two Long Terminal Repeats (LTRs)—and have, thus, the potential for transcriptional activity [1,4]. In addition, a great proportion of these repetitive elements are present in human chromosomes as single LTRs, termed solo LTRs, which have originated from recombination events. This is evident when comparing HERVs integrations in humans and their orthologs in primates [5,6,7]. Nonetheless, both proviral and solo LTRs may retain sequences able to control gene expression, such as promoters, enhancers, and polyadenylation signals [8].

Overall, few HERV insertions have been studied to understand their implication in human pathophysiology [9]. The best-described example of HERV involvement in the host physiology is the production of syncytin-1, a retroviral protein coded by the *env* gene of a provirus belonging to the HERV-W group, expressed in human trophoblasts [10,11,12,13]. Other HERV proviruses and proteins have been investigated for their possible involvement in pathogenesis, in particular, in both cancer and autoimmunity [13]. In fact, there are several pieces of evidence showing an abnormal increase in HERV expression in tumor cells [14,15,16,17,18]. Probably this HERV upregulation is due to the lack of transcriptional control in cancer. In most cases, the transcripts are not translated and overall, there are only a few examples of a causative role of HERVs in tumorigenesis [1,14,19]. The complex connection between HERVs and the immune response has been also widely investigated [20,21]. Indeed, some inflammatory settings can induce HERV expression, while some HERV products may trigger the host immune response, and hence, activate the innate immune pathways [20,21,22,23]. Importantly, extensive knowledge of the mechanisms of HERV-mediated immune activation would be essential for the understanding of possible HERV implications in inflammatory conditions as well as in autoimmune diseases [21,24].

HERVs can also have an impact on human biology other than through protein production [9,25]. HERV LTRs include enhancers, promoters, polyadenylation signals and splice sites within their sequences, and may influence neighboring cellular gene expression [9,25,26]. In addition, HERV integrations may alter the normal gene functions by providing alternative and aberrant sites for splicing or by interfering, either positively or negatively, with its mRNA transcription through the production of non-coding RNAs [27].

Given that HERVs/LTRs represent a large portion of the human genome and can potentially influence our physiology, it is quite clear that cells should finely control HERV transcriptional and translational activity through various mechanisms such as accumulation of mutations, RNA silencing, and histone/DNA methylation [28,29,30,31,32]. While most HERVs are silenced, some elements are normally expressed in various developmental stages of human embryogenesis, and their activity is regulated in different human tissues [31,33,34]. HERVs could be also activated as a consequence of some pathological conditions, like HIV infections or cancer, characterized by alterations in epigenetic regulation [15,35,36,37]. Overall, such expression patterns make it difficult to clearly establish a causal association between HERVs and diseases.

Some of the latest integrated members of the HERV-K HML-2 group are insertional polymorphisms in the human population [38]. The identification of insertional polymorphic proviruses might be important in the investigation of the role of HERVs in human biology, as physiological or pathological phenotypic variants may co-occur with or be associated to such polymorphic HERV insertions [39]. 

The great majority of the studies about the effects of HERVs on human pathophysiology are based on microarrays, hybridization-based approaches, or reverse transcription followed by polymerase chain reaction (RT-PCR). Unfortunately, due to technical limitations, these studies have often failed to explain the complexity of the HERVs impact on host biology in its entirety [40]. However, the sequencing of the human genome, the resulting genomic characterization of HERVs and, finally, the advent of high-throughput technologies has led to a great advancement in this field [41]. In fact, such technologies have allowed one to take into account genome variations, to analyze regulatory elements and the three-dimensional organization of the genome, and to characterize the HERV transcriptome [42,43]. This review focuses on new insights of HERV contribution to human pathophysiology and the development obtained through the application of high-throughput sequencing technologies. We briefly describe the HERV databases currently available, then we discuss the possible applications of specific high-throughput sequencing technologies to the HERV field. Finally, we report an overview of discoveries on the role of HERVs in human biology made through the application of these high-throughput sequencing techniques.

## 2. Identification of HERVs in the Human Genome

The comprehensive sequencing of the human genome has made an important contribution to genetics and HERV research. An initial improvement has been the possibility to identify and classify retroviral sequences through computational methods. One of the most used software for the identification of HERVs and other repetitive elements is RepeatMasker (http://www.repeatmasker.org), a program that checks the genomes for interspersed repeats, by making use of a database of repetitive sequences, Repbase (https://www.girinst.org). RepeatMasker also makes use of Dfam (https://www.dfam.org), another database of repetitive sequences organized by families. Of note, the analysis of RepeatMasker allows for collecting of the majority of repetitive elements, referred to as HERVs and solo LTRs, but it is not able to predict the retroviral structure of HERV proviruses. A similar database, hervgdb4, has been created with the specific aim to detect HERVs through an Affymetrix array (HERV-V3). Hervgdb4 includes proviral and solo LTR sequences that have been collected by using 42 selected proviral sequences (prototypes) as references for RepeatMasker analyses or, alternatively, by reconstructing proviral structures from data of the Dfam database [44]. Of note, the “prototype” subset of sequences in the hervgdb4 database also includes gene annotation. Since this database has been created to design the probes of an Affymetrix array, all the sequences are fragmented [44]. A third interesting tool is RetroTector, a software developed for the automated recognition of the best-preserved proviral sequences in the genome of vertebrates [45,46]. The HERV recognition process through RetroTector led to a related study that analyzed the human genome assembly GRCh 37/hg19, identifying, characterizing and classifying a total of 3173 HERV sequences [3]. The classification work, based on a multiple approach, split the HERV proviruses into 39 well-supported phylogenetic groups, belonging to the retroviral classes I (Gamma- and Epsilon-like), II (Beta-like), and III (Spuma-like) [3]. Interestingly, an additional 31 noncanonical HERV groups were identified, revealing a high degree of mosaicism [3]. RetroTector can also predict the sequence of the retroviral genes and a multitude of other retroviral features, like the Primer Binding Site (PBS) or the Poly Purine Tract (PPT), as well as the putative protein products. By contrast, it is unable to detect solo LTRs [3].

In addition to the mentioned three larger databases, there are also some smaller ones that only collect the HERV sequences belonging to a single group. This kind of database usually starts with the analyses of the human genome with RepeatMasker, RetroTector, or both. The data collected are then manually visualized and inspected, and sometimes implemented by performing BLAT searches. The HERV coordinates provided in these databases are, hence, the most accurate and well-annotated. Example of HERV groups deeply studied are the HERV-W, and several HML subgroups [6,32,47,48,49,50].

The abovementioned HERV databases differ especially in the number of HERV elements included, the accuracy of the coordinates, and the availability of gene annotations and other retroviral features. Therefore, a proper application of high-throughput in HERVs research should start with the choice of the best HERV database for that specific analysis. For example, a large and inclusive dataset may be useful for the identification of HERVs as biomarkers. Instead, the identification of putative physiological or pathological functions, or the prediction of coding capability, may need accurate coordinates and possibly, gene annotations.

## 3. Examples of High-Throughput Applications in HERV Research

Since the first draft of the human genome sequence was completed in 2001 [51,52], thousands of full genome sequences have become publicly available [53]. High-throughput sequencing technologies have allowed the performance of multiple genome and transcriptome sequencing in parallel. For example, DNA sequencing and RNA sequencing (RNA-seq) can help to evaluate human genomic diversity through the identification of variants and mutations [42]. DNA–protein interactions, such as Chromatin Immunoprecipitation sequencing (ChIP-seq) and Methylation sequencing (Methyl-seq), are useful to explain epigenetic changes [43]. At the transcriptomic level, RNA-seq can be instead used to analyze the transcriptome and identify modulated genes, while Ribosome sequencing (Ribo-seq) can determine mRNA transcripts that are being translated [43]. 

Before starting the description of some examples of high-throughput applications in HERV research, it is necessary to underline some limitations of such technologies in this field. Indeed, most RNA-seq data analysis software, which have not been specifically designed to identify repetitive elements, may have some difficulties in properly mapping short-reads to repetitive regions of the genome. For this reason, it is necessary to take into account the limitations of the application of this technology to repetitive sequences, including HERVs [54]. Indeed, the number of HERVs identified after mapping the reads to the genome may be underestimated, as the majority of pipelines do not count reads mapping equally well to two or more genomic regions [54]. Moreover, a good practice is the use of the paired-end and high-quality reads sequences, and to always keep in mind possible complications in de novo assembly of whole genomes and mapping in repetitive regions [55,56,57].

### 3.1. Genome Sequencing and HERVs Variability

**Figure 1:**
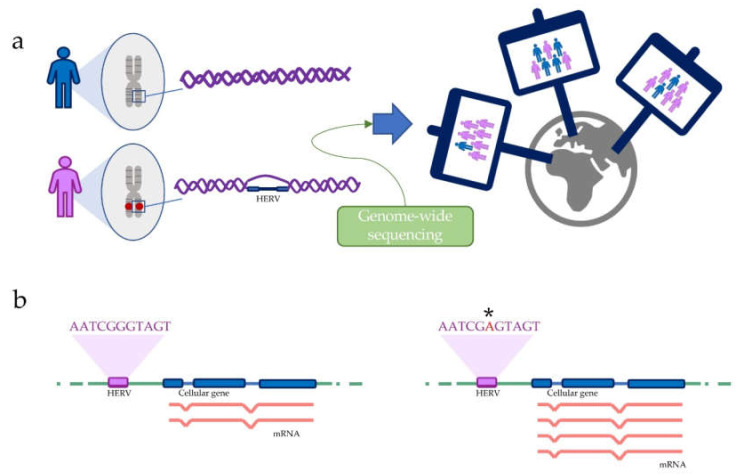
Example of possible application of high-throughput sequencing in the study of Human Endogenous Retrovirus (HERV) variability. The high-throughput sequencing of whole genomes allows the identification of insertional polymorphisms and to assess the genome-wide distribution of these polymorphic loci (**a**). This application also allows the identification of single nucleotide variations associated with expression quantitative trait loci (*), which explain changes in the host gene expression levels (**b**).

As already mentioned, there is a certain HERV variability in the human genome. A deep knowledge of such variability may effectively help to better understand the real impact of HERVs in human biology. For this reason, one of the most essential challenges in HERVs research is the identification of insertional polymorphisms (Figure 1a). For this purpose, several studies take advantage of data coming from large datasets of whole-genome sequences. Indeed, many projects have provided numerous copies of whole-genome sequences in the form of short DNA fragments, namely reads, collected in databases. Whole-genome sequences sources can include different type of data, e.g., data from healthy donors, like those collected by the 1000 Genomes Project [53,58], or from pathological samples, like those from cancer patients collected by The Cancer Genome Atlas (TCGA) [59] and the International Cancer Genome Consortium (ICGC) [60,61]. Reads from these sources can be mapped to a reference human genome assembly, and variation from the latter may be useful to identify polymorphic insertions or single nucleotide changes [42,43] (Figure 1a). Hence, some bioinformatics tools have been developed to discover insertional polymorphic transposable elements, including one that is specifically designed for HERVs [62]. In this way, it is possible to detect insertional polymorphic HERVs and to study their frequency in different human populations [63,64,65,66,67,68]. However, it is still difficult to study insertional polymorphisms within the same individual by using whole-genome sequences data. The application of these technologies remains, hence, insufficient to investigate HERV mobility or somatic integrations [41]. 

The identification of Single Nucleotide Polymorphisms (SNPs) into the HERVs sequence is another application that required whole-genome sequences data, especially for its possible implication in diseases [53,58]. Indeed, SNPs may be associated with Expression Quantitative Trait Loci (eQTLs), which are variants that explain changes in the host gene expression levels (Figure 1b) [39,69,70]. Such variations in gene expression can be accountable for phenotypical or pathological traits, and HERVs, including SNPs associated with eQTLs, can hence be directly linked to physiology and diseases [39,69].

### 3.2. Regulation of HERV Expression and Impact on Human Gene Expression

**Figure 2:**
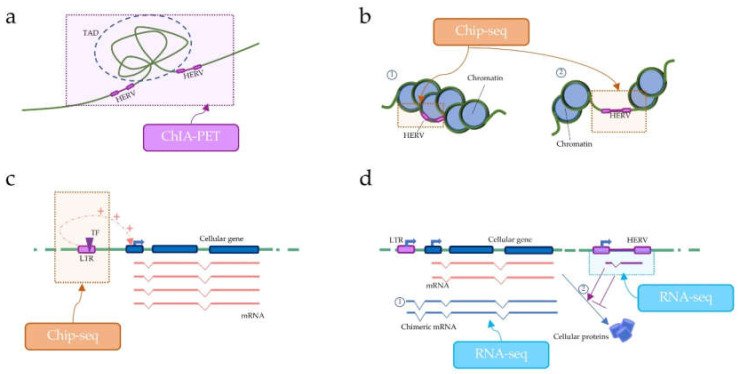
Example of possible application of high-throughput sequencing to the study of HERV regulation of gene expression. Paired-end tag sequencing (ChIA-PET) can be used to analyzed 3D interactions of the chromatin. It is possible to investigate HERV involvement in the formation of Topologically Associating Domains TADs (**a**). Chromatin Immunoprecipitation sequencing (ChIP-Seq) allows the analysis of DNA–protein interactions. ChIP-Seq can be used to check the chromatin state of HERV loci (**b**), which may be transcriptionally silenced (1) or transcriptionally active (2). ChIP-Seq can be also used to study the interaction of DNA with other proteins, e.g., Transcription Factors (TFs). TF–LTRs interactions can enhance the expression of neighbor genes through cis-regulatory mechanisms (**c**). RNA-seq technologies allow the analysis of individual HERV loci expression that may influence cellular gene expression (**d**). For example, HERVs may provide alternative promoters to neighbor genes, resulting in HERV-gene chimeric transcripts (1). HERV non-coding RNAs (ncRNAs) can also be detected through RNA-seq approaches (2).

High-throughput sequencing technologies have profoundly improved our knowledge of the three-dimensional organization of the genome, chromatin state, and chromatin modifications [42]. The 3D chromatin can be analyzed by paired-end tag sequencing (ChIA-PET) and Hi-C [71], which consist of consecutive steps of legation and sequencing of close crosslinked chromatin portions, so that frequently interacting portions can be visualized in the contact matrix [42,71]. These techniques are particularly useful to identify regions of the same chromosome that preferentially interact with each other due to the particular organization of the chromatin in Topologically Associating Domains (TADs). The main application of these techniques in HERV research is the investigation of possible involvements of HERVs in TADs’ formation (Figure 2a) [34,72]. 

The chromatin state and modification can also be studied through the application of ChIP-Seq technologies. Indeed, ChIP-Seq allows the analysis of DNA–protein interactions, through the combination of chromatin immunoprecipitation and high-throughput sequencing [42,43]. When applied to the HERV research, ChIP-Seq can be used to check the chromatin state of HERV loci, and its modification in different developmental stages or diseases (Figure 2b) [73,74]. ChIP-Seq can be also used to study the interaction of DNA with proteins other than those composing chromatin, and to predict, for example, the interaction between LTRs and Transcription Factors (TFs). Indeed, due to the presence of enhancers in their LTR sequences, HERVs and solo LTRs integrated near to a cellular gene can enhance the expression of the gene through cis-regulatory mechanisms (Figure 2c) [21]. 

HERV integrations may also interfere with the normal gene functions by providing alternative promoters, polyadenylation signals, and sites for splicing [21,75,76]. These mechanisms of interference can result in HERV-gene chimeric transcripts, which may be computationally reconstructed by applying RNA-seq to the transcriptome, and then, by using software tools that detect reads mapping in splice junctions [75]. HERV non-coding RNAs (ncRNAs) can also be detected through RNA-seq approaches, and the results obtained could be used as a starting point for studies of HERV-mediated cis-regulation of cellular genes (Figure 2d) [21,77]. 

### 3.3. Identification of Expressed and Modulated HERV Loci

**Figure 3:**
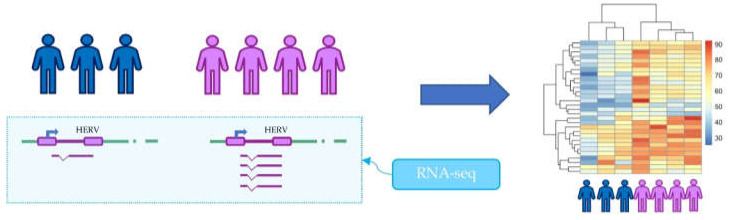
Example of possible application of high-throughput sequencing for the identification of expressed and modulated HERV loci. The application of RNA-seq to the HERV transcriptome can provide the expression levels of the individual loci. Differential expression analyses can show HERVs modulation in different conditions, for example, healthy control versus disease.

The study of HERV role in human pathophysiology has also been performed through the analysis of the expression of each HERV locus and its modulation in different healthy and diseased tissues (Figure 3) [13]. Indeed, the causal relationship between HERVs and diseases is still not clear, and most of the studies have not been able to identify the expression of individual loci, but were limited to the expression of entire HERV families [35,78]. The application of RNA-seq to the HERV transcriptome can provide essential information on the transcriptional contribution of the different families, and especially, the expression levels of the individual HERV loci based on their unequivocal localization in the human genome. For example, RNA-seq pipelines for differential expression have been used to show over- or under-expression of HERVs in tissues [79], diseases [80,81], and medical treatments [82]. The data obtained with this approach can be used as a starting point for understanding the importance of HERV transcripts and their possible translation into proteins, but also to identify HERVs to be used as biomarkers [36]. Finally, bioinformatics pipelines for metagenomics allowed the study of HERV contribution to the human intestinal virome [83]. 

## 4. New Insights on HERV Contribution to Human Pathophysiology and Development

### 4.1. HERV Variability in Human Population

The HERV-K (HML-2) group is known to include some young elements integrated into the genome of modern humans after the divergence from the lineage of chimpanzees (*Pan troglodytes*) and bonobos (*Pan paniscus*) [47]. Moreover, the HML-2 group may have been active in archaic hominids also after the divergence from the lineage of modern humans [84,85]. Indeed, some studies have analyzed the genome sequences of Neandertal and Denisovan, identifying some HML-2 insertions not included in the modern human genome assembly [84,85]. Specifically, Agoni et al. identified 14 HML-2 loci in Neanderthal and Denisovan, including 8 for which coordinates have been clearly detected. Only seven of these insertions in archaic hominids were also found in the genome of certain modern humans, as polymorphic insertions [58,65,84]. Moreover, Lee et al. found six HML-2 integrations that appear to be unique to Neanderthal and Denisovan genomes [84]. Hence, the recent insertional activity of the HML-2 group is of particular interest for the presence of polymorphic proviruses [38,47]. In total, there are 36 HML-2 proviruses in the human population that are not included in the human reference genome [39,63,64,65,84,85]. Furthermore, also some of the proviruses present in the human genome assemblies hg19 and hg38 are known to be unfixed among the population [39,47,64]. The analysis of data from the 1000 Genomes Project has revealed differences in the frequency of HML-2 insertions among the five super-populations: African, East Asian, Admixed American, European, and South Asian. Not all the HML-2 insertions occur in the different populations with the same frequency [63,64], and the state of presence or absence of the totality of the proviruses is sufficient to distinguish the five super-populations [63,64]. Many of the unfixed insertions are rare, and the East Asian population is the one with the lowest prevalence of HML-2 insertional polymorphisms [63,64]. About the potential role of these loci, interesting data derive from the insertions that have significant Single Nucleotide Polymorphism (SNP) association enriched for eQTLs. Indeed, such information tries to establish a relationship between a single nucleotide variant for HML-2 polymorphic and tissue-specific gene expression [39,69]. Interestingly, 46 insertional polymorphisms have SNPs enriched for eQTLs across 44 human tissues [39]. Moreover, 15 of them have SNPs associated with specific neurologic and immunologic traits, including Parkinson’s disease and other autoimmune diseases [39]. 

Polymorphisms in HERVs that are not polymorphic insertions may also be very informative. For example, polymorphisms occurring in transcription factor binding sites may explain the differential HERV expression among individuals and in cancer [86]. Moreover, many HERV elements have been found to be enriched for somatic mutations (hotspots) in cancer [69]. Among these hotspots, the mutation C2270G in ZNF99 is associated with a lower survival rate in kidney cancer patients, and it might be potentially used as a biomarker [69].

### 4.2. HERV Expression is Regulated during Human Development

HERVs are regularly expressed in the germline, but the epigenetic regulation is essential to finely control their expression since the first steps of embryogenesis [31,33,87,88,89]. The HML-2 group was reported to be expressed during the early embryonic stages, in cells from morula and pre-implantation blastocysts [89]. Such proviral RNAs result in the production of retroviral products, such as Gag proteins and the HML-2 accessory protein Rec [89]. The expression pattern of HERV-H elements is a key component of pluripotency, and a useful application is to use their expression as a marker for capturing the human naïve pluripotent state in vitro [74,90]. HERV-H elements provide functional binding sites for transcription factors driving the production of chimeric transcripts that modulate pluripotency acting as long ncRNAs [87,91,92]. For example, HERV-H LTRs can include binding sites for the LTR-binding protein 9 (LBP9) [91,93], NANOG protein [74,88], and the Octamer-Binding Transcription Factor 4 (OCT4) [88]. 

Among the HERV-H group, several proviruses are also transcribed. The classical RNA structure from expressed proviruses is 5′LTR-gag-pro-3′LTR, but seems not to include intact open reading frames. Of note, HERV-H RNA constitutes about 2% of all poly-A RNA in embryonic cells [67]. 

HERV-H elements actively contribute to the creation of transcriptionally active and self-interacting compartments, TADs [34,94]. Importantly, the creation of HERV-mediated TAD boundaries suggests that these elements can have an important impact on gene regulation [72,95].

The repression of HERV expression is then established in the pre-implantation embryo and is maintained in most developed tissues [96]. The mechanisms of HERV silencing vary, and many data refer to murine Endogenous Retroviruses (mERVs) in a mouse model. Firstly, DNA methylation, catalyzed in mice by DNA methyltransferases, is required to repress mERVs in differentiated cells [96,97]. Moreover, the protein Histone-lysine N-methyltransferase SETDB1 may have a critical role in inhibiting mERVs expression, as it is evident in SETDB1 knockout embryonic cells, where several mERVs are de-repressed [98]. Indeed, HERVs may take advantage of the Ten-eleven translocation methylcytosine dioxygenase (TET) class of proteins to evade DNA methylation and transcriptional repression. For this reason, cells may have evolved methylation-independent silencing pathways, like histone modification, during developmental stages or tissues when DNA methylation is compromised [97]. KRAB zinc finger proteins (KZFPs) are also involved in HERV silencing by targeting repressive chromatin states [99,100]. Finally, a microRNA (miR-34a) can repress mERV expression by restraining some transcription factor binding proteins [101].

### 4.3. HERVs Contribute to Somatic Cell Physiology and Disease

HERVs are transcribed also in somatic cells [66,97]. A study analyzed the HERV expression in RNA-Seq samples from the ENCODE project, finding that HERVs are active in a cell line-specific manner [73,79,102]. Of course, as the consequences of HERV activity in somatic cells may be deleterious, the host makes a great effort to efficiently repress the great majority of HERV expression [103,104]. Indeed, 794,972 LTR sequences have Transcription Factor Binding Sites (TFBSs)—most of which co-localized with genes involved in the immune response—that may potentially interfere with neighboring genes [105]. The activity of HERV proviruses and solo LTRs is modulated in response to stress and immune activation [15,106,107]. For example, some MER41 elements include STAT1- and IRF1-binding sites and mediate the activation of the response to pathogens [72,105]. The high number of regulatory elements linked to the immune response is not accidental [108]. In fact, by introducing and amplifying interferon-sensitive enhancers, HERV integrations have shaped the evolution of transcriptional pathways that define the interferon response [108]. In the hippocampus, an area of the brain particularly susceptible to stress, the acute stress is correlated with the silencing of HERVs [107]. HERVs were shown to be also modulated by the histone deacetylases inhibitor vorinostat, which reactivates HIV in latently infected cells [82].

HERVs are known to be de-silenced, and hence, transcriptionally activated in cancer. Accordingly, HERV transcriptional activity has been found to be significantly upregulated in cancer cells than in controls [73,80,86,109,110,111]. 

Patterns of HERV modulation are evident also in other diseases. For instance, some HERV-W and HERV-H loci have been found to be expressed in postmortem brain samples from schizophrenic and bipolar patients [112]. The HERV-W group has been often tentatively correlated to multiple sclerosis [22,23]. Nonetheless, transcript levels of HERV-W loci were similar in healthy brain samples and in multiple sclerosis lesions, suggesting a lack of HERV-W modulation in the presence of the disease [113]. Furthermore, analyses of HERV expression have led to contrasting results in PBMCs from patients with systemic lupus erythematosus. A first work observed in fact a general trend of HERV downregulation [114], while a second one identified 124 significantly upregulated HERV loci and no downregulated ones [57]. HERVs have also been recently associated with drug addiction. This is the case of a polymorphic HML-2 solo LTR found in antisense orientation within the sequence of a gene that affects dopaminergic activity [77]. The expression of this antisense LTR can modulate the expression of the neighbor gene, and this HML2 integration is more frequently present in drug-addicted individuals as compared to the general population [70].

## 5. Concluding Remarks

The application of novel high-throughput sequencing technologies has allowed great improvements in the understandings of the HERV contribution to human physiology and pathology. The application of these technologies gives essential insights about HERV variability in the population, HERV expression and silencing during human development, their involvement in the transcriptional activity of the host, and last but not least, HERV expression and modulation in several diseases. Importantly, different from other techniques, such an approach allows the obtaining of important information about the individual HERV loci, being either polymorphic or fixed in the human population. Of course, the analysis of the transcription allows for the obtaining of data on the HERV expression, and further analysis of translation and proteomics are needed to identify retroviral proteins expressed and their functions.

Overall, high-throughput sequencing has, hence, the great potential to provide all-inclusive bases to understand the impact of HERV in the host biology.
